# Genetic variations in NLRP3 and NLRP12 genes in adult-onset patients with autoinflammatory diseases: a comparative study

**DOI:** 10.3389/fimmu.2023.1321370

**Published:** 2024-01-26

**Authors:** Mark Yun, Zuoming Deng, Brianne Navetta-Modrov, Baozhong Xin, Jie Yang, Hafsa Nomani, Olga Aroniadis, Peter D. Gorevic, Qingping Yao

**Affiliations:** ^1^ Division of Rheumatology, Allergy and Immunology, Stony Brook University Renaissance School of Medicine, Stony Brook, NY, United States; ^2^ Biodata Mining and Discovery Section, National Institute of Arthritis and Musculoskeletal and Skin Diseases, Bethesda, MD, United States; ^3^ Molecular Diagnostics Laboratory, DDC Clinic for Special Needs Children, Middlefield, OH, United States; ^4^ Department of Family, Population and Preventive Medicine, Stony Brook University Renaissance School of Medicine, Stony Brook, NY, United States; ^5^ Division of Gastroenterology and Hepatology, Stony Brook University Renaissance School of Medicine, Stony Brook, NY, United States

**Keywords:** autoinflammatory disease, Cryopyrin-associated periodic syndrome, digenic, genetically transitional disease, mutation, NLRP3, NLRP12, variant

## Abstract

**Objectives:**

Cryopyrin-associated periodic syndrome or NLRP3-associated autoinflammatory disease (NLRP3-AID) and NLRP12-AID are both Mendelian disorders with autosomal dominant inheritance. Both diseases are rare, primarily reported in the pediatric population, and are thought to be phenotypically indistinguishable. We provide the largest cohort of adult-onset patients and compared these diseases and the gene variant frequency to population controls.

**Methods:**

A cohort of adult patients with AIDs were retrospectively studied. All underwent molecular testing for periodic fever syndrome gene panels after extensive and negative workups for systemic autoimmune and other related diseases. Patients were divided into Group 1- NLRP3-AID patients with NLRP3 variants (N=15), Group 2- NLRP12-AID with NLRP12 variants (N=14) and Group 3- both NLRP3 and NLRP12 (N=9) variants. Exome sequence data of two large control populations including the ARIC study were used to compare gene variant distribution and frequency.

**Results:**

All 38 patients were Caucasian with women accounting for 82%. Median age at diagnosis was 41 ± 23 years and the disease duration at diagnosis was 14 ± 13 years. We identified statistically significant differences between the groups, notably that gastrointestinal symptoms as well as evaluations for same were significantly more frequent in patients with NLRP12 variants, and headaches/dizziness were less common among the NLRP12 patients. Livedo reticularis was noted in four patients, exclusively among NLRP12 carriers. Over 50% of patients in Groups 1 and 2 carry low-frequency disease-associated variants, while the remaining carry rare variants. We unprecedently identified digenic variants, i.e., the coexistence of NLRP3 and NLRP12, which were either both low frequency or low frequency/rare. Allele frequencies of all variants identified in our cohort were either absent or significantly lower in the control populations, further strengthening the evidence of susceptibility of these variants to SAID phenotypes.

**Conclusion:**

Our comparative study shows that both NLRP3-AID and NLRP12-AID share similar clinical phenotypes, yet there are significant differences between them with regard to gastrointestinal and neurological symptoms. A spectrum of high to low genetic variations in both genes can contribute to SAID individually or in combination.

## Introduction

Systemic autoinflammatory diseases (SAIDs) primarily affect the innate immune response and are distinct disease entities, though they may overlap somewhat with autoimmune disorders (1). The spectrum of SAIDs has been expanded in recent years (2). Nucleotide oligomerization domain (NOD)-like receptors (NLRs) are a specialized group of intracellular proteins that play a critical role in the regulation of host immune responses (3, 4). Mutations in the NLR family members have been linked to a variety of autoinflammatory diseases (5).

Two examples of NLRs associated with SAIDs are NLRP3 and NLRP12. The NLRP3 gene encodes Cryopyrin, and mutations cause gain-of-function, leading to Cryopyrin-associated Periodic Syndrome (CAPS) (6), aka NLRP3-associated autoinflammatory disease (NLRP3-AID) (5). CAPS encompasses a spectrum of phenotypes ranging from mild Familial Cold Autoinflammatory Syndrome Type 1 (FCAS1), Muckle-Wells syndrome (MWS) to severe Chronic Infantile Neurological Cutaneous and Articular (CINCA), or Neonatal-onset Multisystem Inflammatory Disease (NOMID) (7, 8). NLRP3-AID is typically caused by NLRP3 gene missense mutations of high penetrance; however, an intermediate form has been reported to be associated with a low-penetrance variant, NLRP3 Q705K, aka Q703K (9, 10).

Genetic variations in NLRP12 cause NLRP12-AID (11), aka Familial Cold Autoinflammatory Syndrome Type 2 (FCAS2) due to its similarity to FCAS1 and MWS, and both NLRP12-AID and CAPS are phenotypically characterized by conjunctivitis, urticaria and arthralgias triggered by exposure to cold, as well as neurosensory hearing loss (11). Similarly, NLRP12-AID is associated with NLRP12 missense mutations of high penetrance resulting in gain-of-function; however, nearly 50% of reported cases to date harbor the NLRP12 variant, F402L, a low-penetrance variant (12). To date, molecular testing has been key for differentiating between these two diseases, as they are thought to be clinically indistinguishable. Like CAPS, NLRP12-AID is a rare autosomal dominant disease. There have been approximately 60 cases reported in the literature as of the year 2020 (13). Most reported cases were pediatric. In addition, we have not seen any reports of cases of concurrent NLRP3 and NLRP12 variants. Based on the Infevers database (https://infevers.umai-montpellier.fr/web/search.php?n=4) as of July 24, 2023, total numbers of sequence variants identified for NLRP3 and NLRP12 are 264 and 82, respectively. Both NLRP3-AID and NLRP12-AID are caused by a spectrum of a number of validated known and yet-to-be validated variants. Based on the current literature, both NLRP3-AID and NLRP12-AID are clinically indistinguishable. Herein, we investigated the largest monocentric cohort of adult-onset patients to compare these two diseases in order to identify clinical parameters for distinction. We also examined the frequency of disease-associated variants in two large control datasets to support their contributory roles in these diseases.

## Patients and methods

### Study design, population and genetic testing

This is a retrospective study. A cohort of adult patients with SAIDs were studied. All had multidisciplinary evaluations and were seen at our Center of Autoinflammatory Disease between January 1, 2016 and March 31, 2023. All patients underwent molecular testing by next generation sequence analysis for a 6-gene panel that includes *MEFV, TNFRSF1A, NLRP3*, MVK*, NLRP12* and *NOD2* (DDC Clinic, Middlefield, Ohio, USA). Approximately 27% patients also underwent molecular testing for an extended autoinflammatory gene panel (Invitae, California, USA). An individual SAID was diagnosed based on characteristic phenotype and specific genotype, as well as conformity with classification criteria for periodic fever syndromes (14). Inclusion criteria were adult patients aged 18 and older, and they had autoinflammatory features, notably cold-induced urticaria and/or hearing loss. These patients were diagnosed as NLRP3-AID if *NLRP3* variants were detected or NLRP12-AID if *NLRP12* variants were present. Exclusion criteria were other SAIDs, systemic autoimmune diseases, infections, and inflammatory bowel disease.

### Definitions

The initial description of MWS in 1962 comprised a triad of urticaria, deafness and amyloidosis; however, patients with this classic triad have been rarely reported. There have been no diagnostic criteria available to differentiate MWS and FCAS1 (15). In our study, CAPS patients were defined to have MWS if they had autoinflammatory phenotypes and coexisting sensorineural hearing loss; FCAS1 if they had autoinflammatory phenotypes and cold-induced urticaria without sensorineural hearing loss; and an “intermediate form” if they had autoinflammatory phenotypes without cold-induced urticaria or sensorineural hearing.

### Control datasets

To discover mutations associated with disease, the central premise is that affected persons harbor a significant excess of pathogenic DNA variants as compared with a group of unaffected persons (controls) (16). The most popular approach used by researchers in human genetics is the case–control design (17). To estimate the distribution and frequency of individual and combined variant alleles identified from our patients in control populations, our collaborators at the National Institutes of Health (NIH) used the large database, dbGaP from the Atherosclerosis Risk in Communities (ARIC) study (18) with dbGaP accession number phs000280.v8.p2. The ARIC study’s data are owned by the National Heart Lung and Blood Institute and are publicly available to qualified investigators. The ARIC study includes a cohort population and several community surveillance populations in the United States. ARIC initiated community-based surveillance in 1987 for myocardial infarction and coronary heart disease incidence and mortality and created a prospective cohort of 15,792 Black and White adults aged 45 to 64 years. In our study, there were 2,952 individuals selected based on European-American ancestry and the availability of high-quality whole exome sequencing (WES) data. The individual and combined gene alleles involved in our SAID patients were analyzed in the control population, and their frequency is listed (**Table 1**).

**Table 1:** Frequency of individual and combined gene variants in patients and control.

All groups	Genomic coordinates	Patients (N=38), n (%)	ARIC dbGap control (N=2,952) n (%)	P-value*
Total NLRP3 Q705K	1-247588858-C-A	19(50.00)	322(10.91)	<0.0001
Total NLRP12 F402L	19-54313707-G-C	14(36.84)	470(15.92)	0.0016
NLRP3-AID patients (group 1), N=15**				
Q705K(Q703K)	1-247588858-C-A	10(26.32)	282(9.55)	0.0027
V200M(V198M)	1-247587343-G-A	4(10.53)	53(1.78)	0.0054
A441V(A439V)	1-247588067-C-T	1(2.63)	0(0)	0.0127
NLRP12-AID patients (group 2), N=14***				
F402L	19-54313707-G-C	8(21.05)	433(14.67)	0.2528
P286L	19-54314056-G-A	1(2.63)	1(0.03)	0.0253
A682T	19-54312869-C-T	1(2.63)	0(0)	0.0127
F402L+ A682T		1(2.63)	0(0)	0.0127
A218V	19-54314260-G-A	1(2.63)	0(0)	0.0127
E315K	19-54313970-C-T	1(2.63)	0(0)	0.0127
G448A	19-54313570-C-G	2(2.63)	1(0.03)	0.0005
H304Y	19-54314003-G-A	0(0)	16(0.54)	1.0000
R919W	19-54304482-G-A	0(0)	1(0.03)	1.0000
R206C	19-54314297-G-A	0(0)	0(0)	–
NLRP3 and NLRP12 patients (group 3), N=9#				
NLRP3 Q705K+NLRP12 F402L		6(15.79)	37(1.25)	<0.0001
NLRP3 Q705k + NLRP12 H304Y		1(2.63)	3(0.10)	0.0499
NLRP3 Q705k + NLRP12 R919W		1(2.63)	0(0)	0.0127
NLRP3 Q705K+V200M+ NLRP12 R206C		1(2.63)	0(0)	0.0127

*P-values were from two-sided Fisher’s exact test.

** There were 15 patients with NLRP3-AID, including 7 MWS (5 with Q705K and 2 V200M), 5 FCAS1 (3 with Q705K, 1 A441V and 1 V200M), and 3 intermediate form (2 with Q705K and 1 V200M).

*** There were 14 patients with NLRP12-AID or FCAS2.

# There were 9 patients with both NLRP3 and NLRP12 variants and all had mixed diagnoses(overlaping symptoms) of both NLRP3-AID and NLRP12-AID.

In addition, exome sequencing data of 100 subjects that were Old Order Amish (Caucasian) and aged 18-65 (Research Cohort in Molecular Diagnostics Laboratory, DDC Clinic, Ohio, USA) were used as another reference control. These subjects were either unaffected controls or affected with a rare Mendelian genetic disorder other than SAIDs, noting that neither NLRP3 nor NLRP12-AID has been described in this population. The exome sequencing data were analyzed to examine the distribution and frequency of the NLRP3 and NLRP12 gene variants (**Table 2**).

**Table 2:** Frequency of our patients’ variants in the DDC control and general population.

NLRP3	Genomic coordinates	DDC control (100) MAF or %	Global gnormAD MAF, %
Q705K/Q703K	1-247588858-C-A	8%	3.8% (10781/280716)
V200M/V198M	1-247587343-G-A	0%	0.8% (2351/282566)
A441V/A439V	1-247588067-C-T	0%	0%
NLRP12			
F402L	19-54313707-G-C	3%	5.1% (14333/282762)
P286L	19-54314056-G-A	0%	0.01% (40/282440)
A682T	19-54312869-C-T	0%	0.002% (6/281132)
F402L+ A682T		0%	NA
A218V	19-54314260-G-A	0%	0.0008% (2/251148)
E315K	19-54313970-C-T	0%	0.0004% (1/251304)
G448A	19-54313570-C-G	0%	0.005% (13/281838)
H304Y	19-54314003-G-A	0%	0.4% (1251/282632)
R919W	19-54304482-G-A	0%	0.2% (632/281544)
R206C	19-54314297-G-A	0%	0.008% (21/250696)
NLRP3 and NLRP12			
NLRP3 Q705K+NLRP12 F402L		0%	NA
NLRP3 Q705k + NLRP12 H304Y		0%	NA
NLRP3 Q705k + NLRP12 R919W		0%	NA
NLRP3 Q705K+V200M+ NLRP12 R206C		0%	NA

### Statistical analysis

Kruskal-Wallis test was used to compare continuous variables such as age, and the Chi-square test with exact p value from Monte Carlo simulation was used to compare categorical variables such as sex among three patient groups. In addition, median+/-IQR were reported for continuous variables; column percentages were reported for categorical variables. Fisher’s exact test was used to compare prevalence of different genotypes between Group 1 patients and the control population. To compare the frequency of combined variants with the ARIC data, all raw p-values were reported. The significance level is set at p<0.05 and all analysis was performed using SAS 9.4 (SAS Institute Inc., Cary, NC).

## Results

### Demographic, clinical, and laboratory data of patients in all groups

A total of 38 adult patients with SAIDs were included and divided into three groups: Group 1 (N=15, 39.5%)- NLRP3-AID patients with NLRP3 variants only, Group 2 (N=14, 36.8%)-NLRP12-AID patients with NLRP12 variants only, and Group 3 (N=9, 23.7%)- patients with both NLRP3 and NLRP12 variants only. In Group 1, 7 patients with NLRP3 variants were classified as MWS, 5 FCAS1, 3 intermediate form, and none had the CINCA/NOMID phenotype. Demographic, clinical, and laboratory data are listed in **Table 3**. The median age at disease diagnosis was 41 ± 23 years and disease duration was14 ± 13 years. All patients exhibited autoinflammatory disease phenotypes, i.e., a constellation of complete or partial manifestations, such as recurrent fever, rash, arthralgia, abdominal pain/recurrent diarrhea, chest pain, and hearing loss among others.

**Table 3:** Demographic, clinical and laboratory data of patients and their comparisons among three groups of patients.

Variable	Level	Total (N=38)	NLRP3-AID (N=15)	NLRP12-AID (N=14)	NLRP3+NLRP12 (N=9)	P-value*
Age at diagnosis (year)		41.00 ± 23.00	38.00 ± 24.00	41.00 ± 21.00	44.00 ± 19.00	0.8919
Disease duration at diagnosis (year)		14.00 ± 13.00	15.00 ± 9.00	7.50 ± 17.00	20.00 ± 9.00	0.0445
Sex	Female	31 (81.58%)	10 (66.67%)	12 (85.71%)	9 (100.00%)	0.1492
	Male	7 (18.42%)	5 (33.33%)	2 (14.29%)	0 (0.00%)	
Race	Caucasian	38 (100.00%)	15 (100.00%)	14 (100.00%)	9 (100.00%)	.
Family history			7(46.67%)	6(42.86%)	3(33.3%)	
Fatigue	No	6 (15.79%)	4 (26.67%)	2 (14.29%)	0 (0.00%)	0.2511
	Yes	32 (84.21%)	11 (73.33%)	12 (85.71%)	9 (100.00%)	
Night sweats	No	28 (73.68%)	9 (60.00%)	11 (78.57%)	8 (88.89%)	0.2628
	Yes	10 (26.32%)	6 (40.00%)	3 (21.43%)	1 (11.11%)	
	Yes	13 (34.21%)	8 (53.33%)	1 (7.14%)	4 (44.44%)	
Headache/dizziness	No	22 (57.89%)	5 (33.33%)	12 (85.71%)	5 (55.56%)	0.0176
	Yes	16 (42.11%)	10 (66.67%)	2 (14.29%)	4 (44.44%)	
Fever	No	15 (39.47%)	4 (26.67%)	7 (50.00%)	4 (44.44%)	0.4476
	Yes	23 (60.53%)	11 (73.33%)	7 (50.00%)	5 (55.56%)	
Skin rash	No	6 (15.79%)	4 (26.67%)	1 (7.14%)	1 (11.11%)	0.3967
	Yes	32 (84.21%)	11 (73.33%)	13 (92.86%)	8 (88.89%)	
Arthralgia	No	7 (18.42%)	3 (20.00%)	3 (21.43%)	1 (11.11%)	0.8834
	Yes	31 (81.58%)	12 (80.00%)	11 (78.57%)	8 (88.89%)	
Lower extremity swelling	No	26 (68.42%)	11 (73.33%)	9 (64.29%)	6 (66.67%)	0.9057
	Yes	12 (31.58%)	4 (26.67%)	5 (35.71%)	3 (33.33%)	
Myalgia	No	23 (60.53%)	11 (73.33%)	8 (57.14%)	4 (44.44%)	0.3361
	Yes	15 (39.47%)	4 (26.67%)	6 (42.86%)	5 (55.56%)	
Oral ulcer	No	27 (71.05%)	10 (66.67%)	12 (85.71%)	5 (55.56%)	0.2859
	Yes	11 (28.95%)	5 (33.33%)	2 (14.29%)	4 (44.44%)	
Abd pain	No	21 (55.26%)	11 (73.33%)	8 (57.14%)	2 (22.22%)	0.0615
	Yes	17 (44.74%)	4 (26.67%)	6 (42.86%)	7 (77.78%)	
Diarrhea	No	18 (47.37%)	11 (73.33%)	5 (35.71%)	2 (22.22%)	0.0298
	Yes	20 (52.63%)	4 (26.67%)	9 (64.29%)	7 (77.78%)	
Sicca	No	19 (50.00%)	6 (40.00%)	10 (71.43%)	3 (33.33%)	0.1468
	Yes	19 (50.00%)	9 (60.00%)	4 (28.57%)	6 (66.67%)	
Eyelid swelling	No	26 (68.42%)	10 (66.67%)	9 (64.29%)	7 (77.78%)	0.8194
	Yes	12 (31.58%)	5 (33.33%)	5 (35.71%)	2 (22.22%)	
Hearing loss/decrease	No	29 (76.32%)	10 (66.67%)	12 (85.71%)	7 (77.78%)	0.5607
	Yes	9 (23.68%)	5 (33.33%)	2 (14.29%)	2 (22.22%)	
Asthma	No	28 (73.68%)	12 (80.00%)	9 (64.29%)	7 (77.78%)	0.6429
	Yes	10 (26.32%)	3 (20.00%)	5 (35.71%)	2 (22.22%)	
Proteinuria/hematuria	No	33 (86.84%)	14 (93.33%)	12 (85.71%)	7 (77.78%)	0.6083
	Yes	5 (13.16%)	1 (6.67%)	2 (14.29%)	2 (22.22%)	
Raised ESR/CRP/ferritin	No	27 (71.05%)	8 (53.33%)	11 (78.57%)	8 (88.89%)	0.1587
	Yes	11 (28.95%)	7 (46.67%)	3 (21.43%)	1 (11.11%)	
Hypogammaglobinemia	No	35 (92.11%)	13 (86.67%)	14 (100.00%)	8 (88.89%)	0.4309
	Yes	3 (7.89%)	2 (13.33%)	0 (0.00%)	1 (11.11%)	
Chest pain	No	29 (76.32%)	10 (66.67%)	11 (78.57%)	8 (88.89%)	0.4541
	Yes	9 (23.68%)	5 (33.33%)	3 (21.43%)	1 (11.11%)	
Pleuritis	No	34 (89.47%)	13 (86.67%)	12 (85.71%)	9 (100.00%)	0.6561
	Yes	4 (10.53%)	2 (13.33%)	2 (14.29%)	0 (0.00%)	
Pericarditis	No	34 (89.47%)	12 (80.00%)	14 (100.00%)	8 (88.89%)	0.2616
	Yes	4 (10.53%)	3 (20.00%)	0 (0.00%)	1 (11.11%)	

*: For continuous variables, p-values were based on Kruskal–Wallis tests; for categorical variables, p-values were based on Chi-squared test with exact p-value from Monte Carlo simulation.

In addition, median+/-IQR were reported for continuous variables; column percentages were reported for categorical variables.

### Clinical similarities and differences between NLRP3-AID and NLRP12-AID

In comparing the patient groups with regard to clinical manifestations and laboratory data, we found statistically significant differences in several aspects. First, gastrointestinal (GI) symptoms (recurrent abdominal pain and diarrhea) occurred in all three groups but the latter was significantly more frequent among patients with NLRP12 or digenic NLRP3/NLRP12 variants than those with NLRP3 variants alone. Diarrhea was similarly frequent without significant difference between patients with NLRP12 ± NLRP3 variants. Some patients underwent more detailed gastrointestinal evaluations, including endoscopy/colonoscopy, which were performed more frequently in NLRP12 carriers with 53% (N=8) in NLRP12, 67% (N=6) in NLRP3/NLRP12, and only 13% (N=2) in the NLRP3 group. For those who underwent in-depth GI workups, there was no imaging, endoscopic, or pathological evidence of inflammatory bowel disease. Second, there was a statistically significant difference in neurological complaints, including headaches/dizziness, among the three groups, with 67%(N=10) NLRP3, 44% (N=4) NLRP3/NLRP12, and 7%(N=1) NLRP12 incidences. In addition, some patients underwent extensive neurologic workups without specific findings, though a few individuals carrying NLRP3 variants were noted to have striking bilateral hand tremors, and one had a history of seizures. Third, the nature of cutaneous presentations differed between groups. Livedo reticularis was only seen among NLRP12 (N=2) or digenic NLRP3/NLRP12 (N=2) patients, but not in the largest NLRP3 group, though patchy erythema was seen in all groups. Hearing loss was identified across all groups. Thirty percent of patients in all three groups had elevated acute-phase reactants (ESR and/or CRP), with no significant difference between the three. A full plasma cytokine panel 13 (IL-2R, IL-12, IFN, IL-4, IL-5, IL-10, IL-13, IL-1β, IL-6, IL-8, TNFα, IL-2, IL-17) was performed in 6 patients, including 5 in NLRP12 group with elevated IL-10 levels seen in 4. The cytokine panel was tested in one patient in the NLRP3/NLRP12 group, who was also found to have elevated IL-10.

### Genotyping data of patients in all groups

The gene variants identified in all three patient groups are listed in **Tables 1**, **2**. Of the 15 NLRP3-AID patients with NLRP3 variants only in group 1, 10 (67%) carried Q705K (aka Q703K), 4 (27%) with V200M (akaV198M), and 1 (7%) with A441V (pathogenic). Of the 14 NLRP12-AID patients with NLRP12 variants only in group 2, 8 (57.1%) carried F402L, 1 with P286L, 1 with F402L/A682T, 1 with A218V, 1 with E315K, and 1 with G448A (pathogenic). In NLRP3/NLRP12 mixed group, 56% (N=5) carried Q703K+F402L variants. Other variants identified in our patients are rare. There were no other autoinflammatory disease gene variants identified using the periodic fever syndrome 6-gene panel among the 38 patients.

In genomic medicine, it is important and useful to examine the frequency of an individual gene variant in the general population as an approach to distinguish benign from possibly pathogenic variants (13). To compare the frequency of these individual variants and their combinations in patients with that in control populations, we used the large ARIC dataset (2,952 subjects) as a control and computed the Minor Allele Frequencies (MAFs) of the NLRP3 Q705K and NLRP12 F402L to be 0.049 and 0.076, respectively. Only a very low percentage (1.186%, 35/2,952) of subjects carried both NLRP3 Q705K and NLRP12 F402L. In sum, we found that individual and combined gene variants were significantly more frequent among our patients than the control. In other words, most variants were completely absent in the control population. In addition, our collaborators at DDC Clinic, OH used their research cohort of 100 Amish samples and computed the MAF to be 0.08 and 0.03 for NLRP3 Q705K and NLRP12 F402L, respectively, although neither SAID has yet to be identified or reported in this special population. Most importantly, no patient among the research cohorts carried combined NLRP3 and NLRP12 variants or the rare variants identified in our patients.

### Therapeutic responses to IL-1 inhibitors among the three groups

Ten out of 15 patients (67%) with NLRP3 variants were treated with IL-1 inhibitors, with patient-reported symptomatic improvement relative to the baseline (**Table 4**). Of the 14 patients with NLRP12 variants, only 21% (N=3) were treated with IL-1 inhibitors, and 2 patients reported improvement, whereas 1 patient was resistant. Among the NLRP3/NLRP12 digenic group, 56% (N=5) were treated with IL-1 inhibitors, of whom, 3 responded and 2 did not improve.

**Table 4:** Therapeutic responses to IL-1 inhibitors.

Patient	Sex	Age at Dx	Genotype	Diagnosis	IL-1 inhibitor	Dosage	Frequency	Efficacy Improvement	Side effects	Treatment duration	Continuing use
1	F	32	NLRP3, A441V	FCAS1	Can	150 mg	Every MTH	100%	Thrombocytopenia	6 MTH	No
2	M	34	NLRP3, Q705K	Int	Can	150 mg	Every MTH	100%	None	12 MTH	Yes
3	F	25	NLRP12, V200M	Int	Can	150 mg	Every MTH	60%	Thrombocytopenia	6 MTH	No
4	F	56	NLRP3, Q705K	MWS	Can	150 mg	Every 2 MTH	100%	None	6 yrs	Yes
5	F	37	NLRP3, Q705K	MWS	Can	150 mg	Every MTH	100%	None	6 yrs	Yes
6	F	33	NLRP12, V200M	FCAS1	Anakinra	100 mg	Daily	80%	None	3 yrs	Yes
7	F	35	NLRP12, V200M	MWS	Anakinra	100 mg	Daily	50%	None	1 yr	Yes
8	M	38	NLRP3, Q705K	MWS	Can	150 mg	Every MTH	100%	None	5 yrs	Yes
9	F	46	NLRP3, Q705K	FCAS1	Can	150 to 300 mg	Every MTH	50%	None	3 yrs	Yes
10	M	22	NLRP3, Q705K	FCAS1	Can	150 mg	Every MTH	50%	None	1 yr	No
11	F	43	NLRP12, F402L	NLRP12-AID	Anakinra	100 mg	Daily	100%	Transient injection site reaction	8 MTH	Off 9 MTH, asymptomatic
12	F	25	NLRP12, F402L	NLRP12-AID	Can	150 mg	One dose	No	Pneumonia		No
13	F	58	NLRP12, P.Ala218Val	NLRP12-AID	Can	150 mg	Every 3 MTH	No	None	9 MTH	No
14	F	56	NLRP3 Q705K+NLRP12 F402L	Mix	Can	150 mg	Every 2 MTH	50%	Weight gain	1 yr	No
15	F	48	NLRP3 Q705K+NLRP12 F402L	Mix	Can	150 mg	Every 2 MTH	90%	Pneumonia	2 yrs	No
16	F	38	NLRP3 Q705K+NLRP12 F402L	Mix	Can	150 mg	Every 2 MTH	90%	None	2 yrs	Yes
17	F	32	NLRP3 Q705K+NLRP12 F402L	Mix	Can	150 mg	Every MTH	No	None	2 MTH	No
18	F	46	NLRP3 Q705K, NLRP2 V200M, NLRP12 p.Arg206Lys	Mix	Can	150 mg	Every MTH	No	Sinusitis	2 MTH	No

FCAS1, Familial cold autoinflammatory syndrome 1; Can, canakinumab; Int, intermediate; MWS, Muckle-Wells syndrome; MTH, month.

## Discussion

NLRP3-AID and NLRP12-AID are autosomal dominant disease and patients share similar phenotypes, including cold-induced urticaria/inflammatory disease and neurosensory hearing loss. Genotyping, therefore, has been key for their distinction (11, 12). In the present study, patients in both Group 1 and 2 had a family history of the disease in approximately 45% (**Table 3**). We found statistically significant differences in certain clinical phenotypes among disease groups. In NLRP3-AID, complaints of headache and dizziness were significantly more common (P value=0.0176), consistent with a previous report of 79% of CAPS patients with known pathogenic NLRP3 variants and 73% of those with low-penetrance variants (9). We also found that GI symptoms (diarrhea) in patients with NLRP12-AID were significantly more common than among the NLRP3 group. In a previous report, only 25% of patients with pathogenic NLRP3 variants reported abdominal pain and none diarrhea (9). In support of our finding are two previous reports that a CAPS mouse transgenic model carrying human NLRP3 pathogenic mutations maintain homeostasis in the gut microbiota and are resistant to experimental colitis (19), whereas NLRP12 defect results in microbiome disturbance and colitis (20). In our study, hearing loss occurred infrequently across all groups with no significant difference (33% patients with NLRP3 variants). In contrast, sensorineural hearing loss was reported for 79% of patients with NLRP3 pathogenic variants and 18% with NLRP3 low-penetrance variants (9), indicating that mutations of high-penetrance may be more pathogenic for hearing loss. In addition, we noted that four patients had livedo reticularis and this occurred only among NLRP12 carriers. Taken together, our data suggest that these clinical features can be used for distinguishing these two diseases.

Over 60% of our patients in Group 1 carry the low-penetrance variant, NLRP3 Q705K, which is known to increase risk for CAPS (9). Compared with the large ARIC data, the variant frequency was significanly higher in our patient group (**Table 1**). NLRP3 is primarily involved in inflammasome activation leading to production of proinflammatory cytokines like IL-1β, and its mutations cause gain-of-function leading to NLRP3-AID. In a multicenter study of 45 patients with low penetrance NLRP3 variants including 19 with Q705K vs 28 CAPS patients with known pathogenic variants, the former patients had autoinflammatory features, such as fever (76%), headaches (73%), rash (80%), sensorineural hearing loss (18%), arthralgia (84%), abdominal pain (56%), diarrhea (18%), and elevated ESR/CRP(26%-34%) (9); these clinical findings and their frequencies are quite similar to those in our patients with NLRP3 variants (**Table 3**). *In vitro* studies confirmed that cells expressing NLRP3 Q705K exhibited mildly increased caspase 1 activity and cleavage, and also that these patients responded to IL-1 inhibition therapy (9). In another study, human monocytic cell lines transduced with Q705K produced a significant higher level of IL-1β and IL-18 than wild type, confirming the gain-of-function (21). In agreement with the literature, 67% of our patients with NLRP3 variants received IL-1 inhibitors with a good to excellent response. Taken together, both our and literature data strongly support that autoinflammatory features can be caused by the low-penetrance NLRP3 variants. Of note, compared with patients with low-penetrance variants, those with known pathogenic NLRP3 variants (A439V, E311K and T348M) had a lower frequency of fever (18%) and GI symptoms (0-25%), and a higher frequency of sensorineural hearing loss (79%) (9).

Over 50% of our patients in Group 2 carry the low-penetrance variant, NLRP12 F402L, which is linked to NLRP12-AID. In fact, 44% to 50% of reported cases carried the NLRP12 variant (12, 13). Symptomatic patients with F402L can be children and adults. In a review of 33 pediatric cases, females accounted for 65%, and major clinical manifestations included recurrent fever in 100% of patients, polyarthralgias in 55%, abdominal pain/diarrhea in 48%, rash in 45%, neurosensory hearing loss in 21%, headaches in 24%, and elevated CRP/ESR in 55% (13, 22). These manifestations and frequencies are similar to our findings in adult-onset patients with NLRP12-AID (**Table 3**). To date, there have been few case-control studies to estimate the MAF of NLRP12 F402L. One study involved 5 patients vs 11 F402L carriers among 94 healthy subjects (MAF 0.05) (23) and the other involved 6 patients vs zero F402L carriers among 72 healthy subjects (MAF 0) (24). Apparently, these studies had sample sizes too small to more accurately estimate MAF or determine the effect size of this variant. To overcome the shortcomings of the small sample sizes, we compared our patients with the large ARIC data, and found that the frequency of the variant was significantly lower in the control as compared with patient’s group, supporting the previously reported role of NLRP12 F402L in the disease, NLRP12-AID. While we have not seen functional studies of NLRP12 F402L variant, bioinformatic analysis points to its pathogenicity (13). In addition, one of our patients carried two NLRP12 variants, A682T and F402L. Supporting the clinical significance of this finding is a recent report of a symptomatic adult female with carriage of two heterozygous NLRP12 variants, R352C/F402L inherited from both parents (13).

NLRP12 can negatively regulate pro-inflammatory signaling and inflammation (11, 25, 26). Wild type NLRP12 has a pro-inflammatory effect on caspase 1 signaling and speck formation, induces the release of the inflammasome-dependent cytokines IL-1β and IL-18, and has anti-inflammatory effect on NF-kB activation. It has been reported that the NLRP12 R352C missense mutation is associated with a gain-of-function with regard to caspase 1 processing (27, 28). In our current study, few cases of NLRP12-AID received IL-1 inhibitors with inconsistent effectiveness, by contrast to patients with NLRP3 variants. The mechanisms underlying the therapeutic differences between the two diseases are unclear. The negative regulation of proinflammatory cytokines by NLRP12 suggests that its function may be inflammasome-independent (26). This will need further translational study using larger cohorts of patients.

It is well known since the completion of the human genome project in 2003 (29) that many genetic variants that contribute to disease lie on a spectrum from rare alleles with large effect sizes to more common alleles with small effect sizes. There is a “gray zone” between these two extremes, such as NLRP3 Q705K and NLRP12 F402L in our study, that is ill-defined with regard to terminology, classification and clinical reportability (30). To supplement the traditional interpretation for monogenic autosomal dominant or recessive diseases, we recently proposed and defined the concept “Genetically Transitional Disease” (GTD). GTD refers to a disease status between monogenic and polygenic, where a mutation is necessary, but not sufficient, to cause disease (31). This concept has been used to certain rheumatic diseases (32). Autoinflammatory diseases with low frequency/penetrance variants as in our study are good examples. The GTD concept and related nosology may help with molecular laboratory reports of genetic testing results by inclusion of some “benign variants” or “variants of uncertain significance” (VUS) and inform genetic counselling (33).

Nine patients in our study carry concurrent NLRP3 and NLRP12 variants, including NLRP3 Q703K with NLRP12 F402L in 6/38 (15.8%) patients. This digenic combination was significantly more frequent than the ARIC control (1.25%) and was not identified in the DDC cohort control. We recently reported the contributions of variant combinations of NOD2 and other SAID genes to mixed NLR-associated SAID and their implications (34). To date, we have not seen any reports of the variant combination, NLRP3/NLRP12 only in the literature. Our current data suggest that the variant combination may contribute to digenic disease, where combinations of variants in two genes are required for disease expression (35). In addition, our study has provided new information on the frequency of this digenic combination in control populations as there have been no such data available from the general population to date. Our study indicates that GI involvement may be used to distinguish between NLRP3-AID and NLRP12-AID. Indeed, GI symptoms in CAPS with *NLRP3* mutations are rare likely due to homeostasis maintenance from T regulatory cells induced by the remodeling of intestinal microbiota from hyperactive NLRP3 (19). By contrast, *NLRP12* mutations can cause more GI symptoms as in our current study. Under normal conditions NLRP12 interacts with NOD2, blocking NOD2 signaling. *NOD2* mutations can cause GI symptoms as in Crohn disease and Yao syndrome (36). NLRP12 deficiency induces tolerance of bacterial muramyl dipeptide (MDP), a specific stimulator to initiate NOD2 signaling pathway (37).

In our present study, GI symptoms appear more common in patients with NLRP3/NLRP12 variants than those with NLRP12 variants only, suggesting a synergistic role of both NLRP3 and NLRP12 variants in causing GI disturbance. Regarding the potentially differential impact of *NLRP12* mutations and the digenic variants on disease phenotypes and expressivity, further study using a larger sample size is warranted. To our knowledge, there are no basic or translational studies of potential NLRP3-NLRP12 interaction in autoinflammatory diseases. However, in a human and murine study of infection using peripheral blood mononuclear cells, malaria induced the assembly of ASC, NLRP3 and NLRP12 inflammasomes jointly and promoted cleavage of procaspase-1, leading to IL-1β and hypersensitivity to bacterial superinfection. This study may favor a potential interaction between NLRP3 and NLRP12 under autoinflammatory state particularly with regard to infectious triggers (38). We also noted that compared with patients with NLRP3 variants, plasma cytokine profile was more frequently ordered or tested among patients with NLRP12 variants, with IL-10 levels frequently elevated. Interestingly, both wild type NLRP12 protein and IL-10 are anti-inflammatory (39), suggesting that there could be synergism of these two molecules in autoinflammatory regulation and control.

There are limitations in our study. This is a single center retrospective study with the benefit of uniformity and standardization of the study population seen in an Autoinflammatory Disease Referral Center. Relative to studies of common diseases, the sample size is small due to investigation of rare diseases or clinical scenarios. For the potential role of combined variants from different genes in SAIDs, we previously assumed that the composite effects could be synergistic, antagonistic, or both. This will need further functional study in the future.

## Conclusion

In summary, our study has provided a new insight into the distinction of NLRP3-AID and NLRP12-AID. GI symptoms seem more common in NLRP12-AID and headaches/dizziness seem less common than NLRP3-AID. We also provide further evidence of the diagnostic utility of some genetic variants. For patients with suspected SAIDs, low penetrance variants should not be overlooked when molecular testing results are reported or interpreted. Unlike many common diseases for which there are readily available guidelines or consensus, SAIDs are rare diseases as in our study, and best evidence may come from case reports and case series (40).

## Data availability statement

The original contributions presented in the study are included in the article/supplementary material. Further inquiries can be directed to the corresponding author.

## Ethics statement

The studies involving humans were approved by Stony Brook University Institutional Review Board (approval number IRB2021-00042, initial approval date February 3, 2021). The studies were conducted in accordance with the local legislation and institutional requirements. The ethics committee/institutional review board waived the requirement of written informed consent for participation from the participants or the participants’ legal guardians/next of kin because this is a retrospective study.

## Author contributions

MY: Data curation, Formal analysis, Investigation, Methodology, Resources, Validation, Writing – review & editing. ZD: Data curation, Formal analysis, Investigation, Methodology, Resources, Validation, Writing – review & editing. BN-M: Investigation, Resources, Writing – review & editing. BX: Data curation, Investigation, Methodology, Resources, Validation, Writing – review & editing. JY: Data curation, Formal analysis, Investigation, Methodology, Resources, Validation, Writing – review & editing. HN: Investigation, Methodology, Resources, Writing – review & editing. OA: Resources, Writing – review & editing. PG: Methodology, Writing – review & editing. QY: Conceptualization, Data curation, Formal analysis, Investigation, Methodology, Project administration, Resources, Supervision, Validation, Visualization, Writing – original draft, Writing – review & editing.
